# Neuroserpin polymorphisms and stroke risk in a biracial population: the stroke prevention in young women study

**DOI:** 10.1186/1471-2377-7-37

**Published:** 2007-10-25

**Authors:** John W Cole, Adam C Naj, Jeffrey R O'Connell, Oscar C Stine, John D Sorkin, Marcella A Wozniak, Barney J Stern, Manuel Yepes, Daniel A Lawrence, Laurie J Reinhart, Dudley K Strickland, Braxton D Mitchell, Steven J Kittner

**Affiliations:** 1 Veterans Affairs Medical Center, Baltimore, Maryland, USA; 2 Department of Neurology, University of Maryland School of Medicine, Baltimore, Maryland, USA; 3 Bloomberg School of Public Health, Johns Hopkins University, Baltimore, MD, USA; 4 Department of Epidemiology and Preventive Medicine, University of Maryland School of Medicine, Baltimore, Maryland, USA; 5 Department of Medicine, University of Maryland School of Medicine, Baltimore, Maryland, USA; 6 Department of Neurology, Emory University School of Medicine, Atlanta, GA, USA; 7 Department of Internal Medicine, University of Michigan Medical School, Ann Arbor, MI, USA; 8 Center for Vascular and Inflammatory Diseases, University of Maryland School of Medicine, Baltimore, Maryland, USA

## Abstract

**Background:**

Neuroserpin, primarily localized to CNS neurons, inhibits the adverse effects of tissue-type plasminogen activator (tPA) on the neurovascular unit and has neuroprotective effects in animal models of ischemic stroke. We sought to evaluate the association of neuroserpin polymorphisms with risk for ischemic stroke among young women.

**Methods:**

A population-based case-control study of stroke among women aged 15–49 identified 224 cases of first ischemic stroke (47.3% African-American) and 211 age-matched control subjects (43.1% African-American). Neuroserpin single nucleotide polymorphisms (SNPs) chosen through HapMap were genotyped in the study population and assessed for association with stroke.

**Results:**

Of the five SNPs analyzed, the A allele (frequency; Caucasian = 0.56, African-American = 0.42) of SNP rs6797312 located in intron 1 was associated with stroke in an age-adjusted dominant model (AA and AT vs. TT) among Caucasians (OR = 2.05, p = 0.023) but not African-Americans (OR = 0.71, p = 0.387). Models adjusting for other risk factors strengthened the association. Race-specific haplotype analyses, inclusive of SNP rs6797312, again demonstrated significant associations with stroke among Caucasians only.

**Conclusion:**

This study provides the first evidence that neuroserpin is associated with early-onset ischemic stroke among Caucasian women.

## Background

**Figure 1:**
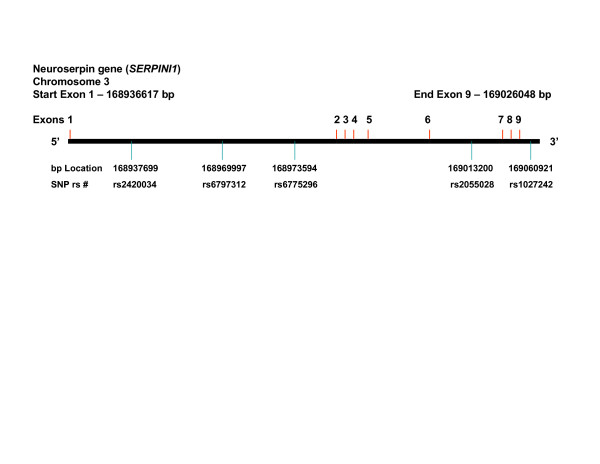
Neuroserpin gene (*SERPINI1*) gene structure demonstrating locations of the SNPs evaluated.

Neuroserpin is a serine protease inhibitor (serpin) that selectively inhibits tissue plasminogen activator (tPA) within the central nervous system (CNS) [1]. Neuroserpin is secreted by neurons in the brain, and provides regulation of tPA activity during both normal and pathological processes including CNS development, neuronal survival, and cerebral ischemia [2,3]. Furthermore, inhibition of tPA activity by neuroserpin protects the barrier function of the neurovascular unit during cerebral ischemia [4] and also plays an important role in the development synaptic plasticity [5-7]. In contrast to its role within the CNS, neuroserpin is not known to interact with tPA in blood where the primary serpin regulating tPA's fibinolytic activity is thought to be plasminogen activator inhibitor 1 (*PAI-1*; OMIM:173360). The human neuroserpin gene (*SERPINI1*; OMIM: 602445), as seen in Figure 1, spans 89.8 Kb on chromosome 3q26.1 and contains 9 exons and 8 introns, producing an mRNA 1159 basepairs in length. The neuroserpin protein consists of 410 amino acids (46.4 kDa) and has 2 primary structural features: 1) a reactive center loop that consists of 16 amino acids which acts as a substrate for tPA, and; 2) a β-sheet that is involved in the permanent deactivation of tPA through the induction of an irreversible conformational change within the tPA protein [7]. Specific mutations in the human neuroserpin gene are known to result in a form of autosomal dominant inherited dementia that is characterized by the presence of intraneuronal inclusion bodies, called Familial Encephalopathy with Neuroserpin Inclusion Bodies (FENIB; OMIM: 604218) [8-10].

A growing body of evidence indicates that the interaction between tissue-type plasminogen activator (tPA) and neuroserpin regulates not only the permeability of the neurovascular unit but also the fate of the ischemic tissue in response to the ischemic insult [2-4,11,12]. Indeed, following experimental middle cerebral artery occlusion (MCAO) there is a rapid increase in tPA activity and neuroserpin expression in the astrocyte-endothelial cell interface [2,4]. Either genetic deficiency of tPA or its inhibition with neuroserpin results in a significant decrease not only in the volume of the ischemic lesion but also in the permeability of the blood-brain barrier [4]. Furthermore, it has been demonstrated that treatment with neuroserpin following ischemic stroke, or overexpression of the neuroserpin gene, results in a significant decrease in the volume of the ischemic area as well as in the number of apoptotic cells [2,3]. Thus, it is highly plausible that endogenous neuroserpin may influence whether or not transient ischemia results in an ischemic stroke.

These findings led us to hypothesize that specific variants in the neuroserpin gene may provide improved or diminished protection with respect to the risk of ischemic stroke. To test this hypothesis, we genotyped several common neuroserpin single nucleotide polymorphisms (SNPs) and tested genotypic and haplotypic association with stroke in a previously collected case-control sample of ischemic stroke among young women.

## Methods

### Study subjects

The Stroke Prevention in Young Women Study 2 (SPYW2) is a population-based case-control study that was designed to examine genetic risk factors for ischemic stroke in young women. The term "population-based" indicates that the cases and their comparison group are representative of the general population and were identified from the same defined population, which included all of Maryland (except the far Western panhandle), Washington DC, and the southern portions of both Pennsylvania and Delaware. Cases included 239 female patients 15 to 49 years of age with a first cerebral infarction as identified by discharge diagnosis at 52 regional hospitals and through direct referral by regional neurologists. The methods for discharge surveillance, chart abstraction, and case adjudication have been described previously [13-15]. The adjudication of stroke cases was performed blinded to genetic information. Stroke cases were classified as having a probable, possible or undetermined etiology as per prior description [13,14]. We excluded fifteen subjects from our analyses based on a modification of the exclusion criteria used in the Siblings With Ischemic Stroke Study (SWISS) [16] protocol: sickle cell disease (1), CNS vasculitis by angiogram and clinical criteria (3), post-radiation arteriopathy (1), endocarditis (3), neurosyphillis (1), mechanical prosthetic heart valves (2), left atrial myxoma (1), and cocaine use in the 48 hours prior to their stroke (3). Control subjects were 212 women without a history of stroke, identified by random-digit-dialing and were frequency matched to the cases by age and geographic region of residence. One control was excluded from analyses based on a history of sickle cell disease. Thus, the sample for genetic analyses consisted of 224 cases and 211 controls.

Cases and controls were grouped into the following race and ethnic categories: Caucasian (non-Hispanic) (n = 95 cases and 99 controls), African-American (n = 105 cases and 91 controls), and other (including Hispanic, Asian, American-Indian, etc.) (n = 24 cases and 21 controls). Because of the small size and heterogeneity of the latter group, it was not analyzed separately, but was included in analyses on the combined total study group (n = 224 cases and 211 controls). Haplotypic association analyses were conducted on only the Caucasian (non-Hispanic) and the African-American groups. Strokes were further classified by subtype; the atherosclerotic group included 27 cases with either probable or possible atherosclerotic mechanism; the cardiac group included 14 cases with a probable cardiac source of embolism; the probable dissection group included 13 cases confirmed by neuroimaging; the lacunar group included 45 cases of symptomatic small deep lesions on neuroimaging studies or classic lacunar syndromes regardless of other potential causes; and the probable hematologic group included 9 cases. These categories were not mutually exclusive. There were 125 non-lacunar stroke cases of undetermined etiology.

Ethical approval for the study was obtained from the University of Maryland School of Medicine Institutional Review Board.

### SNP selection

In an effort to eliminate redundancy in the information provided by the SNPs genotyped, a set of haplotype tagging SNPs were identified through HapMap [17] for both African-Americans and Caucasians. To represent our Caucasian population we identified SNPs that capture 80% of haplotype diversity among European Caucasians. These SNPs were then "force included" to determine the additional tagging SNPs needed fromYorubans to capture 80% of haplotype diversityin our African-American population. A minor allele frequency of >.05 was required. After removing markers with minor allele frequency <0.05, we prioritized the remaining markers by: 1) non-redundant haplotype tagging coverage among both races, 2) distribution throughout the gene, and 3) maximizing the allele frequencies for both SNP alleles. On this basis six SNPs were genotyped in our stroke cohort, including: rs2420034, rs6797312, rs6775296, rs2055028, rs1027242, and rs13090569. Complete HapMap coverage was not attained.

### Genotyping methods for the case/control population

Genotyping was conducted using DNA isolated from whole blood using the QIAamp DNA Blood Maxi Kit (Qiagen, Valencia, CA). SNP genotyping was performed by the Taqman method (Applied Biosystems, Foster City, CA). This method is based on four primers, two flanking the SNP that are used to amplify the DNA surrounding the SNP and two that were labeled with different fluorescent dyes, one for each alternative allele. The original form of the labeled primer has a quencher in close proximity to the dye. When the 5'→3' exonuclease activity of DNA polymerase disrupts the primer hybridized to the single strand DNA during the PCR, the quencher and dye are released, producing a measurable level of fluorescence. The reaction protocol was specified in manufacturer's instructions included with each individual primer set.

### Analyses

All statistical analyses were performed using SAS^®^, Version 9.1 (SAS Institute, Cary, NC). We compared means by two-sided t-tests and proportions by χ^2 ^tests. For those SNPs that satisfied Hardy-Weinberg equilibrium, additive, dominant and recessive models were used to test the effect of genotype on stroke risk. Analyses were conducted in the total population, and also in the African-American and Caucasian women separately. Single SNP association analyses were not adjusted for multiple comparisons because so few SNPs were evaluated and our study was considered to be hypothesis-generating.

Adjusted odds ratios from logistic regression were used to determine whether the presence of the risk allele was associated with an increased risk for stroke after controlling for potential confounders. The minimally adjusted model included age and race. The environmental model included age, race, current cigarette smoking, and oral contraceptive pills (OCP) use. In addition to the covariates of the environmental model, the environmental/vascular risk factor model was adjusted for hypertension, diabetes mellitus, and history of angina or myocardial infarction (angina/MI). Age, race, current cigarette smoking status, and OCP use were based on self-report by subjects (or proxies, if a participant was unable to answer). Hypertension and diabetes mellitus were determined by asking study participants (or a proxy) if a physician had ever told them they had the condition.

Within each ethnic group, analyses were stratified by standard risk factors (age, OCP use, current cigarette smoking, hypertension, diabetes mellitus, and history of angina or myocardial infarction) and stroke subtype (atherosclerotic, cardiac, dissection, lacunar, hematologic, and stroke of undetermined etiology (all other stroke)).

Patterns of linkage disequilibrium (LD) were determined for each race group (African-Americans and Caucasians) using pairwise estimates of D' as implemented in Haploview [18]. Race-specific haplotypic association analyses were performed using Haplo.stats [19] in the statistical software package [20]. Individual haplotypes were estimated in the HaploStats package using the *haplo.em *program, which infers haplotypes and makes haplotype assignments using a progressive insertion algorithm based on an Expectation-Maximization approach, as implemented in the "SNPHAP" software package [21]. Haplotypes first underwent race-specific non-adjusted analyses to evaluate for association with stroke using the haplo.score program. Consistent with the SNP analyses, significant haplotypes were analyzed using age-adjusted, environmental and environmental/vascular models, as well as analyses stratified by stroke subtype using the haplo.glm program. Empirical p-values were estimated for haplotypic association tests in *haplo.score *using a simulation-based approach, thus correcting for multiple comparisons.

## Results

### Subject characteristics

Demographic and risk factor characteristics by case-control status are described in Table 1. The mean age of the cases was 41.7 years and the mean age of control subjects was 39.6 years. Cases were significantly more likely to have a history of hypertension (p < 0.0001), diabetes (p = 0.0002), angina/MI (p = 0.0005), to currently smoke cigarettes (p < 0.0001), and to report the use of oral contraceptive pills (OCP) within the month (31 days) prior to their stroke (p = 0.032).

**Table 1:** Characteristics by case-control status.

	**Cases (N = 224)**	**Controls (N = 211)**	**p-value**
Mean age (years)	41.7	39.6	0.0026
African-American (%)	47.3	43.1	0.579
Hypertension (%)	41.1	14.2	<.0001
Diabetes mellitus (%)	17.9	6.2	0.0002
Current smokers (%)	47.8	23.7	<.0001
Angina/MI (%)	11.6	2.8	0.0005
OCP (%)*	12.2	6.2	0.032

* Two cases and one control could not recall their last OCP use, therefore cases N = 222 and controls N = 210.

### SNP association analyses

Table 2 lists the genotyped SNPs as ordered by their physical position within the gene, and additionally provides the allelic variants, gene region and minor allele frequencies among cases and controls as stratified by race. The five SNPs listed in Table 2 were in Hardy-Weinberg equilibrium. SNP rs13090569 was not in Hardy-Weinberg equilibrium and was therefore excluded from further analyses.

**Table 2:** SNPs analyzed in complete case-control study population including allelic variants, position, gene region, genotype call rate, and minor allele frequencies as stratified by race and case/control status.

**SNP rs number – Alleles**	Position* – Region	**Call Rate**	Allele Frequency**/N			
			**African-American**		**Caucasians**	

			**Cases**	**Controls**	**Cases**	**Controls**

rs2420034 – A/**T**	168937699 – Intron 1	85%	0.17/99	0.11/85	0.49/69	0.45/85
rs6797312 – **A**/T	168969997 – Intron 1	98%	0.43/104	0.42/89	0.64/93	0.56/89
rs6775296 – A/**G**	168973594 – Intron 1	92%	0.14/94	0.19/82	0.10/93	0.07/89
rs2055028 – A/**G**	169013200 – Intron 6	98%	0.24/106	0.23/88	0.10/92	0.14/96
rs1027242 – C/**T**	169060921-3' untranslated	83%	0.48/86	0.44/76	0.47/78	0.47/83

* Relative position (168936217–168936617 exon 1; NCBI Build 35).

** As listed by minor allele frequency in African-Americans (bolded in first column above)

### SNPs associated with stroke

Analyses of the overall study population implementing the 3 inheritance models adjusted for only age and race did not demonstrate any SNP to be associated with stroke (results not shown). However, the A allele (frequency; Caucasian = 0.56, African-American = 0.42) of SNP rs6797312 located in intron 1 (see Figure 1) was associated with stroke in an age-adjusted dominant model (AA and AT vs. TT) among Caucasians (OR = 2.05, p = 0.023) but not African-Americans (OR = 0.71, p = 0.387). Adjusting for other risk factors demonstrated an increased strength of association among Caucasians in both the environmental (OR = 2.53, p = 0.007) and environmental/vascular models (OR = 2.50, p = 0.012). No association was seen among African-Americans in either of the more rigorously adjusted models. These results are summarized in Table 3. Stratifying SNP rs6797312 by risk factors and stroke subtypes revealed no significant associations.

**Table 3:** SNP rs6797312 – Stratified by race in dominant model. Age-adjusted, environmental, and environmental/vascular models demonstrating adjusted odds ratios, 95% confidence intervals, and p-values

**Race**	**Age-Adjusted**			Environmental Model *			Environmental/Vascular Model **		
	OR	95% CI	p-value	OR	95% CI	p-value	OR	95% CI	p-value

African-American	0.71	0.33–1.54	0.387	0.71	0.33–1.56	0.265	0.43	0.18–1.06	0.065
Caucasians	2.05	1.10–3.80	0.023	2.53	1.29–4.95	0.007	2.50	1.22–5.05	0.012

* Adjusted for age, smoking, and oral contraceptive (OC) use.

** Adjusted for age, smoking, OC use, hypertension, diabetes, and angina.

Note: For both the environmental and environmental/vascular models two cases and one control could not recall their last OCP use, therefore cases N = 222 and controls N = 210.

### Haplotype analyses

**Figure 2:**
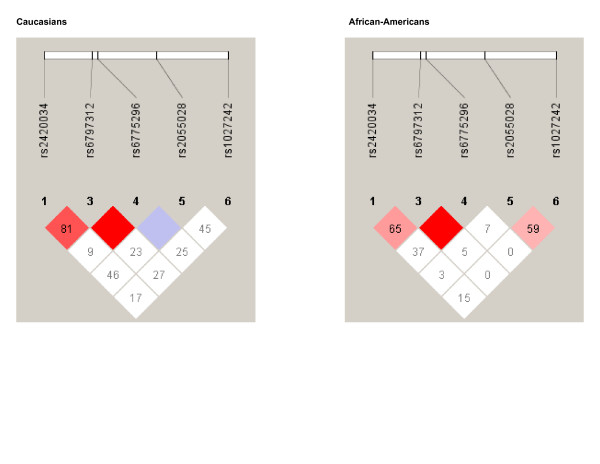
Haplotype block structure among Caucasians and African-Americans.

Figure 2 shows the LD pattern observed among African-Americans and Caucasians. Within both race groups, a haplotype block including SNPs rs6797312 and rs6775296 was observed. Additionally, relatively strong linkage disequilibrium was seen among Caucasians between SNPs rs6797312 and rs2420034. Based upon these observations, we chose to perform race-specific haplotype analyses among Caucasians to include two two-SNP haplotype blocks including rs6797312–rs6775296 and rs24240034–rs6797312, and the three SNP haplotype block rs24240034–rs6797312–rs6775296. Among African-Americans, the two-SNP haplotype block rs6797312–rs6775296 was evaluated.

Among Caucasians, the 3 SNP haplotype rs24240034(A)-rs6797312(A)-rs6775296(A) (haplotype frequency = 25%) was associated with stroke (p = 0.021) in an unadjusted model when evaluating all strokes combined. These associations persisted after controlling for other vascular risk factors in age-adjusted (p = 0.019), environmental (p = 0.004), and environmental/vascular (p = 0.015) models respectively. The stroke of undetermined etiology subgroup demonstrated a trend toward association when analyzed in the unadjusted model (p = 0.10), age-adjusted (p = 0.10), environmental (p = 0.032), and environmental/vascular (p = 0.032) models respectively. Neither of the 2 SNP haplotypes demonstrated associations with stroke.

Among African-Americans, no rs6797312–rs6775296 haplotype demonstrated an association.

## Discussion

Although several studies have demonstrated that neuroserpin plays a role in cerebral ischemia [2-4,22], our study is the first to demonstrate that specific neuroserpin SNPs and haplotypes are associated with the risk of ischemic stroke. While the primary function of neuroserpin appears to be inhibition of tPA in both diseased and non-diseased states [1], increasing evidence indicates that the tPA-neuroserpin axis plays a major role in the regulation of neurovascular unit permeability during cerebral ischemia [2-4,22]. Hence, the beneficial or detrimental role of tPA in cerebral ischemia is location dependent, with intravascular tPA promoting fibrinolysis, and extravascular tPA promoting blood-brain barrier disruption and/or neurotoxicity as regulated by neuroserpin. Furthermore, animal studies of induced ischemic brain injury have demonstrated that the administration of exogenous neuroserpin decreases these effects, hence playing a neuroprotective role, as evidenced by smaller stroke volumes [4]. Thus, it is logical to hypothesize that specific variants in the neuroserpin gene may influence whether or not transient ischemia results in ischemic stroke.

Our study indicates that a specific neuroserpin SNP (rs6797312), and haplotypes including this SNP, are associated with ischemic stroke risk in Caucasian women. The haplotype associations seen in the race-stratified all ischemic stroke groups and the stroke of undermined etiology subtype, suggest that neuroserpin variants may offer varying detrimental or protective effects on ischemia that may be global in nature. Therefore, one could cautiously interpret our results to indicate that SNP rs6797312, or more likely that a nearby SNP in linkage disequilibrium with SNP rs6797312, acts to tilt the tPA-neuroserpin axis towards a less favorable state regarding stroke risk or neuroprotection.

Our study has several limitations. First, our study does not provide any mechanistic information regarding stroke risk or protection. Specifically, we did not determine if our SNPs were associated with increased or decreased neuroserpin activity. Secondly, our study population is relatively small and could be underpowered to detect modest effects, particularly in the stroke subtype analyses. Additionally, even though we evaluated a small set of tagging SNPs identified using LD patterns in HapMap data; the tagging SNPs did not capture all variation in the gene among both race subgroups. Lastly, although we performed numerous analyses, no correction was made for multiple comparisons among the single SNP association analyses, allowing for the possibility that our results could be attained through chance alone. However, the estimation of empirical p-values for haplotypic association tests involving SNP rs6797312 accounts for the issue of multiple comparisons, making a false positive association in haplotype analyses less likely.

## Conclusion

This study provides the first evidence that neuroserpin is associated with early-onset ischemic stroke among Caucasian women.

## Competing interests

The author(s) declare that they have no competing interests.

## Authors' contributions

All authors certify that they participated in the conceptual design of this work, the analysis of the data, and the writing of the manuscript to take public responsibility for it. All authors reviewed the final version of the manuscript and approve it for publication. J.W.C., J.R.O., B.D.M., and S.J.K. participated in the writing of the initial draft. O.C.S. participated in the genotyping. J.W.C., A.C.N., J.R.O., B.D.M., J.D.S., and L.J.R. participated in the data analysis. All authors provided critiques of the final manuscript.

## Pre-publication history

The pre-publication history for this paper can be accessed here:



## References

[B1] Hastings GA, Coleman TA, Haudenschild CC, Stefansson S, Smith EP, Barthlow R, Cherry S, Sandkvist M, Lawrence DA (1997). Neuroserpin, a Brain-associated Inhibitor of Tissue Plasminogen Activator Is Localized Primarily in Neurons. J Biol Chem.

[B2] Yepes M, Sandkvist M, Wong MK, Coleman TA, Smith E, Cohan SL, Lawrence DA (2000). Neuroserpin reduces cerebral infarct volume and protects neurons from ischemia-induced apoptosis. Blood.

[B3] Cinelli P, Madani R, Tsuzuki N, Vallet P, Arras M, Zhao CN, Osterwalder T, Rulicke T, Sonderegger P (2001). Neuroserpin, a neuroprotective factor in focal ischemic stroke. Mol Cell Neurosci.

[B4] Yepes M, Sandkvist M, Moore EG, Bugge TH, Strickland DK, Lawrence DA (2003). Tissue-type plasminogen activator induces opening of the blood-brain barrier via the LDL receptor-related protein. J Clin Invest.

[B5] Krueger SR, Ghisu GP, Cinelli P, Gschwend TP, Osterwalser T, Wolfer D, Sonderegger P (1997). Expression of neuroserpin an inhibitor of tissue plasminogen activator, in the developing and adult nervous system of the mouse. J Neurosci.

[B6] Miranda E, Lomas DA (2006). Neuroserpin: a serpin to think about. Cell Mol Life Sci.

[B7] Yepes M, Lawrence DA (2004). Neuroserpin: a selective inhibitor of tissue-type plasminogen activator in the central nervous system. Thromb Haemost.

[B8] Davis RL, Shrimpton AE, Carrell RW, Lomas DA, Gerhard L, Baumann B, Lawrence DA, Yepes M, Kim TS, Ghetti B, Piccardo P, Takao M, Lacbawan F, Muenke M, Sifers RN, Bradshaw CB, Kent PF, Collins GH, Larocca D, Holohan PD (2002). Association between conformational mutations in neuroserpin and onset and severity of dementia. Lancet.

[B9] Lomas DA, Carrell RW (2002). Serpinopathies and the conformational dementias. Nat Rev Genet.

[B10] Davis RL, Holohan PD, Shrimpton AE, Tatum AH, Daucher J, Collins GH, Todd R, Bradshaw C, Kent P, Feiglin D, Rosenbaum A, Yerby MS, Shaw CM, Lacbawan F, Lawrence DA (1999). Familial encephalopathy with neuroserpin inclusion bodies. Am J Pathol.

[B11] Benchenane K, Berezowski V, Ali C, Fernandez-Monreal M, Lopez-Atalaya JP, Brillault J, Chuquet J, Nouvelot A, MacKenzie ET, Bu G, Cecchelli R, Touzani O, Vivien D (2005). Tissue-type plasminogen activator crosses the intact blood-brain barrier by low-density lipoprotein receptor-related protein-mediated transcytosis. Circulation.

[B12] Berger P, Kozlov SV, Cinelli P, Kruger SR, Vogt L, Sonderegger P (1999). Neuronal depolarization enhances the transcription of the neuronal serine protease inhibitor neuroserpin. Mol Cell Neurosci.

[B13] Johnson CJ, Kittner SJ, McCarter RJ, Sloan MA, Stern BJ, Buchholz D, Price TR (1995). Interrater reliability of an etiologic classification of ischemic stroke. Stroke.

[B14] Kittner SJ, Stern BJ, Feeser BR, Hebel R, Nagey DA, Buchholz DW, Earley CJ, Johnson CJ, Macko RF, Sloan MA, Wityk RJ, Wozniak MA (1996). Pregnancy and the risk of stroke. N Engl J Med.

[B15] Kittner SJ, Stern BJ, Wozniak M, Buchholz DW, Earley CJ, Feeser BR, Johnson CJ, Macko RF, McCarter RJ, Price TR, Sherwin R, Sloan MA, Wityk RJ (1998). Cerebral infarction in young adults: the Baltimore-Washington Cooperative Young Stroke Study. Neurology.

[B16] Meschia JF, Brown RD, Brott TG, Chukwudelunzu FE, Hardy J, Rich SS (2002). The Siblings With Ischemic Stroke Study (SWISS) protocol. BMC Med Genet.

[B17] The International HapMap Consortium (2003). The International HapMap Project. Nature.

[B18] Barrett JC, Fry B, Maller J, Daly MJ (2005). Haploview: analysis and visualization of LD and haplotype maps. Bioinformatics.

[B19] Schaid DJ, Rowland CM, Tines DE, Jacobson RM, Poland GA (2002). Score tests for association between traits and haplotypes when linkage phase is ambiguous. Am J Hum Gen.

[B20] R Development Core Team (2005). R: A language and environment for statistical computing.

[B21] Clayton D SNPHAP – A program for estimating frequencies of large haplotypes of SNPs.

[B22] Zhang Z, Zhang L, Yepes M, Jiang Q, Li Q, Arniego P, Coleman TA, Lawrence DA, Chopp M (2002). Adjuvant treatment with neuroserpin increases the therapeutic window for tissue-type plasminogen activator administration in a rat model of embolic stroke. Circulation.

